# MosChito rafts as a promising biocontrol tool against larvae of the common house mosquito, *Culex pipiens*

**DOI:** 10.1371/journal.pone.0295665

**Published:** 2023-12-14

**Authors:** Agata Negri, Giulia Pezzali, Simone Pitton, Marco Piazzoni, Paolo Gabrieli, Federico Lazzaro, Valentina Mastrantonio, Daniele Porretta, Cristina Lenardi, Silvia Caccia, Claudio Bandi, Sara Epis

**Affiliations:** 1 Department of Biosciences, University of Milan, Milan, Italy; 2 Pediatric Clinical Research Center “Romeo ed Enrica Invernizzi”, University of Milan, Milan, Italy; 3 Italian Malaria Network, Inter University Center for Malaria Research, University of Milan, Milan, Italy; 4 Department of Physics, University of Milan, Milan, Italy; 5 Department of Biomedical, Surgical and Dental Sciences, University of Milan, Milan, Italy; 6 Department of Environmental Biology, “La Sapienza” University of Rome, Rome, Italy; Camerino University: Universita degli Studi di Camerino, ITALY

## Abstract

Mosquito control is of paramount importance, in particular, in light of the major environmental alterations associated with human activities, from climate change to the altered distribution of pathogens, including those transmitted by Arthropods. Here, we used the common house mosquito, *Culex pipiens* to test the efficacy of MosChito raft, a novel tool for mosquito larval control. MosChito raft is a floating hydrogel matrix, composed of chitosan, genipin and yeast cells, as bio-attractants, developed for the delivery of a *Bacillus thuringiensis israeliensis* (*Bti*)-based bioinsecticide to mosquito larvae. To this aim, larvae of *Cx*. *pipiens* were collected in field in Northern Italy and a novel colony of mosquito species (hereafter: Trescore strain) was established. MosChito rafts, containing the *Bti*-based formulation, were tested on *Cx*. *pipiens* larvae from the Trescore strain to determine the doses to be used in successive experiments. Thus, bioassays with MosChito rafts were carried out under semi-field conditions, both on larvae from the Trescore strain and on pools of larvae collected from the field, at different developmental stages. Our results showed that MosChito raft is effective against *Cx*. *pipiens*. In particular, the observed mortality was over 50% after two days exposure of the larvae to MosChito rafts, and over 70–80% at days three to four, in both laboratory and wild larvae. In conclusion, our results point to the MosChito raft as a promising tool for the eco-friendly control of a mosquito species that is not only a nuisance insect but is also an important vector of diseases affecting humans and animals.

## Introduction

Mosquitoes have coexisted with mankind for thousands of years, and several species have impacted the health and the evolution of humans, in relation to their role as disease vectors. Among them, *Culex pipiens* Linnaeus 1758, is the most common mosquito species worldwide and is an important vector of several pathogens, both in the tropics and in the temperate zones [1] as including West Nile virus, Japanese encephalitis virus and lymphatic filariae [2, 3]. Its wide distribution in the urban environments in most continents, including Europe, Asia, Africa and the Americas [4], motivates the epithets of “common house mosquito” and “Northern house mosquito”.

The term *Cx*. *pipiens* refers to a polytypic species, or complex, that includes four species *sensu stricto*: *Cx*. *pipiens*, *Cx*. *quinquefasciatus*, *Cx*. *australicus* and *Cx*. *globocoxitus* [5]. Among them, *Cx*. *pipiens* is further divided into two well-defined forms (or biotypes), one that is typically observed aboveground (*Cx*. *pipiens pipiens*) and the other-one belowground (*Cx*. *pipiens molestus*). These two biotypes are indistinguishable at the morphological level, but very different in their ecology. In terms of physiology and behavior, *Cx*. *pipiens pipiens* is predominantly ornithophilic, feeds and rests outdoors, and requires large spaces to swarm and mate, as well as a blood meal to oviposit the first time; on the contrary, *Cx*. *pipiens molestus* prefers to feed on mammals, including humans, feeds and rests indoors, and is adapted to confined spaces without the need for a blood meal to complete the first oviposition [6].

In Italy and Europe, mosquito control programs are carried out mostly with targeted, local interventions (e.g., municipal or regional), in air against adults and in water against larvae [7]. The developmental phase that is most easily managed is the larval stage, as larvae live in specific and restricted habitats, such as small water pools and containers that represent the breeding sites [7, 8]. The containment of larval populations reduces the number of adult individuals that can transmit pathogens, outdoor and indoors, and mitigate public and environmental concerns due to the spraying of chemical products such as adulticides, in urban and peri-urban areas [8–10]. Larval control ranges from the reduction/elimination of breeding sites to the use of various insecticides (including bioinsecticides) [8, 11, 12]. Larval management can be implemented under the control of public or private administrations, with medium/wide-scale interventions and citizen involvement. For autonomous, citizen-based applications, the use of safe, eco-compatible and easy-to handle products is strongly recommended [13–15].

Insecticides against immature stages, considering their use in water and their longer permanence into the environment, must have two fundamental characteristics: high eco-compatibility and specificity of action against the target species. At present, only a limited number of compounds targeting mosquito larvae have successfully met the requirements of the European Union biocide legislation: compounds from the class of insect growth regulators (IGRs), like products targeting chitin synthesis (e.g., diflubenzuron), and bioinsecticides, based on use of *Bacillus thuringiensis israeliensis* (*Bti*) or *Bti* in combination with *Lysinibacillus sphaericus* (*Ls*) [7, 10]. These microbial bioinsecticides are generally preferred because they lead to immediate and selective death of the larvae, without consequences in terms of pollution; in addition, resistance in target species has been rarely documented [16–19]. On the contrary, resistance to chitin synthesis inhibitors appears to be more common in insects and has also been reported in natural populations of *Cx*. *pipiens* in Italy [20, 21]. It is indeed well-known that improper use of insecticides, even for the most efficient compounds, may lead to the overstimulation of larval defenses and to the selection of resistant individuals [22–24].

To counteract the selection of resistance towards bacterial larvicides, delivery systems are required, to protect these bioinsecticides, enhancing their activity and avoiding their use at sublethal doses, due to degradation in the environment (e.g., under sun-light exposure, water pH, and microbial degradation). Several formulations based on *Bti* are commercially available (e.g., VectoBac^®^12AS), but some of these still present high biodegradability and, therefore, a low duration of action [25, 26]. Two recent studies [27, 28] proposed the use of a delivery system for a *Bti*-based formulate, obtained by inclusion of this bioinsecticide into a hydrogel matrix composed of chitosan (that we called MosChito raft). MosChito rafts indeed had been previously tested [28], under laboratory and semi-field conditions, for both its larvicidal activity against *Aedes albopictus* mosquito larvae, through the embedded *Bti* formulate, and for its phagostimulant potential through the embedded *Saccharomyces cerevisiae* yeast. These studies have led to highly satisfactory results for *Ae*. *albopictus* larval control, in relation to the long duration of the killing action. In the present work, we determined the efficacy of MosChito rafts, both in laboratory and semi-field conditions, on *Cx*. *pipiens*, a mosquito species that often coexists and competes with *Ae*. *albopictus* for the same breeding sites [29, 30].

## Materials and methods

### Mosquito collection, colony establishment, and rearing

Experiments were performed on *Cx*. *pipiens* larvae from a laboratory strain, and on wild-collected larvae. The laboratory strain of *Cx pipiens* was established for the purpose of this study and was obtained from mosquitoes collected in Trescore Balneario (province of Bergamo, Italy) in 2020. Experiments were performed on larvae after about 20 generations in the insectarium at the Department of Biosciences (University of Milan). Briefly, mosquitoes were maintained in the insectarium in accordance with the habits of the species (24 ± 1°C, 45%-50% relative humidity, 12:12 hours light/dark photoperiod). Larvae were fed with granular fish food (Tetra-fish, Melle). Oogenesis and oviposition were made possible by feeding adult females on turkey blood. Wild larvae were collected in the field, at the botanical garden “Cascina Rosa” at the University of Milan (45°28’31.3 "N 9°14’04.3 "E) between June and September 2022. A subsample of the mosquitoes collected for colony establishment and for the experiments on the wild larvae, were identified by morphological keys and PCR-based gene amplification and sequencing, according to published protocols [31].

### MosChito rafts production

Hydrogels rafts were prepared as previously described [27, 28]. MosChito rafts are dishes of 1.6 cm (diameter) × 0.5 cm (thickness), composed of chitosan crosslinked with genipin, containing air bubbles to enable them to float. Two types of rafts were produced: (i) control rafts, composed of chitosan and genipin; and (ii) test rafts containing cells of *Saccharomyces cerevisiae* (strain SY2080; 10^7^ cells/raft) and the *Bti*-based bioinsecticide product, VectoBac^®^12AS (Sumitomo Chemicals Italia SRL, Valent Biosciences). Each MosChito raft contained the commercial product VectoBac^®^12AS at a final concentration of 420 μl/ml. The bioinsecticide concentration was selected, based on previous data collected on *Ae*. *albopictus* [28] (also see next paragraph).

### Bioassays under laboratory conditions

Since MosChito rafts are expected to control the two co-inhabiting species (i.e., *Cx*. *pipiens* and *Ae*. *albopictus*), preliminary bioassays were designed to evaluate whether the VectoBac^®^12AS concentration, previously used in MosChito rafts that were effective against *Ae*. *albopictus* [27], may be also suitable for *Cx*. *pipiens* control. To this purpose, Trescore strain larvae were exposed to the bioinsecticide at a LC_50_ (lethal concentration that causes 50% mortality) determined for *Ae*. *albopictus* (Levate strain, recently established as Trescore strain, see [28]). Tests were performed in accordance with the World Health Organization guidelines [32]. Briefly, pools of 25 4^th^ instar *Cx*. *pipiens* (Trescore strain) or *Ae*. *albopictus* larvae (Levate strain) were transferred into 100 ml of tap water and exposed to 0.370 mg/l of VectoBac^®^12AS. The bioinsecticide was not added to controls. No food was provided, and alive and dead larvae were counted after 24 h. Bioassays were carried out in the insectarium under standard rearing conditions and were repeated at least three times, as independent biological replicates, with at least three groups of larvae for *Ae*. *albopictus* and *Cx*. *pipiens*, for each condition.

### Bioassays under semi-field conditions

**Fig 1:**
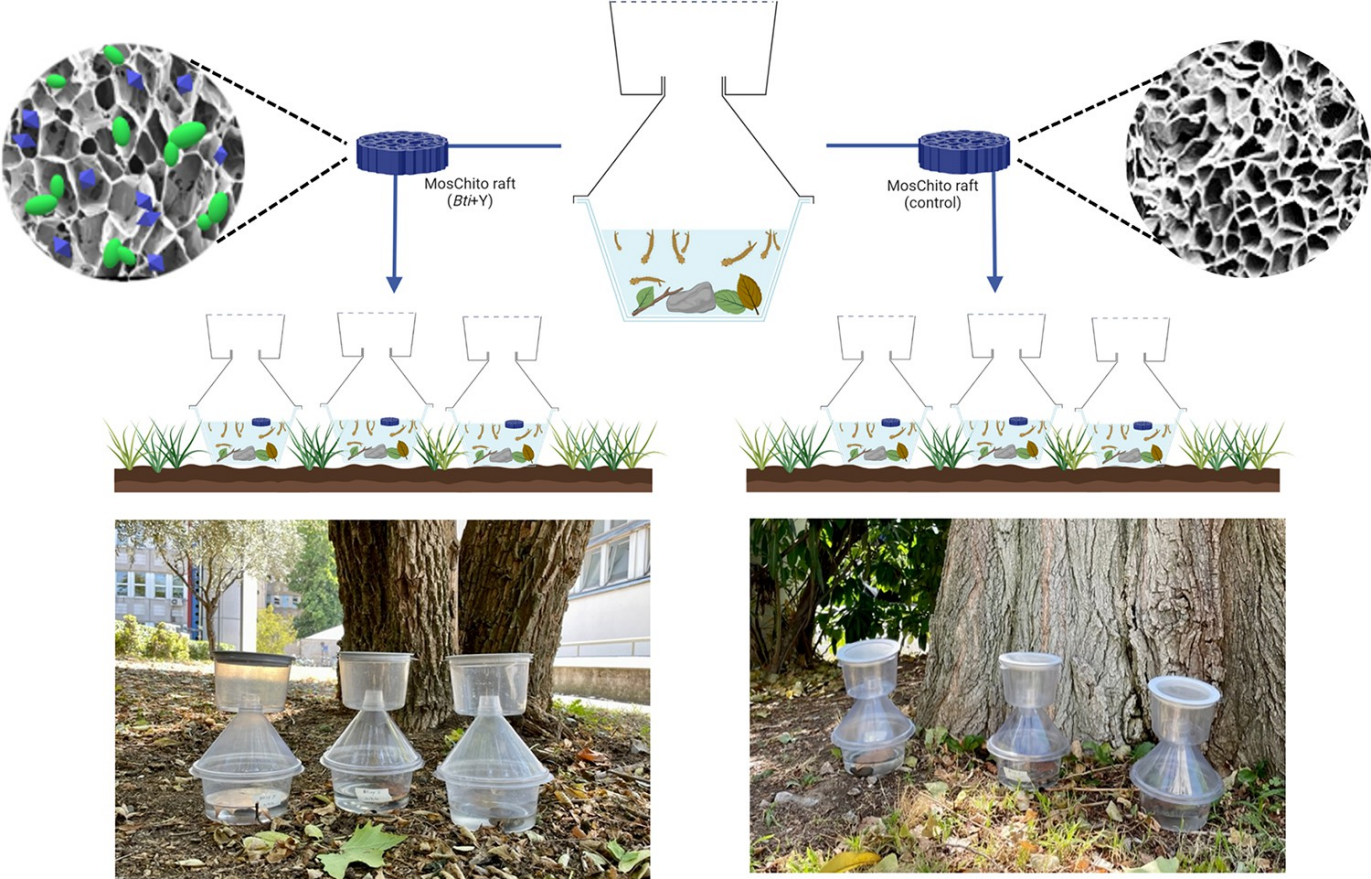
Bioassay in semi-field conditions. Graphical representation and pictures showing the bioassays in semi-field conditions used for *Culex pipiens* larvae exposed to MosChito rafts during summer 2022. Three breeders with *Bti*+Y MosChito rafts and three breeders with control rafts were placed in the backyard of the Department of Biosciences (University of Milan), directly on the ground under the trees and monitored every day, to count alive and dead larvae. Pictures are reprinted from PloSONE under a CC BY license, with permission from BioRender.com, original copyright 2023.

Experiments to evaluate the insecticidal activity of MosChito rafts were performed as follows. Semi-field experiments were performed in the backyard of the Department of Biosciences of the University of Milan (45°28’35.4 "N 9°14’02.9 "E), in the period between June and August 2022. A breeding environment comparable to that observable in peri-domestic areas was recreated in the insect breeders (Bug Dorm provided by NHBS GmbH) (Fig 1), supplemented with 200 ml of rainwater and environmental enrichments (pebbles, leaves, and sand), in which 50 *Cx*. *pipiens* larvae at different developmental stages (from 1^st^ to 4^th^ instar larvae) were placed. Each bioassay included: (i) three breeders with MosChito raft *Bti*+Y containing the commercial *Bti*-based product VectoBac^®^12AS and *S*. *cerevisiae* yeast (Y); (ii) three breeders with the control raft (empty). Alive and dead larvae counts were performed every 24 h, until all treated larvae in the breeders died or pupated. Bioassays were performed separately on laboratory Trescore strain larvae and on wild-collected larvae; experiments were repeated three times.

### Data analysis

Mortality data obtained from laboratory and semi-field assays were analyzed using GraphPad Prism (GraphPad Software Inc. version 8.0). Larval survival of the different strains in the laboratory assays was statistically analyzed by the Student’s *t* test, while the semi-field bioassays were analyzed by the Log-rank (Mantel-Cox) test and between-group comparison, adjusted by FDR (false discovery rate).

## Results

### Mosquito identification by PCR analysis

Mosquitoes from the colony that were reared in the laboratory since 2020 (Trescore strain) and a subsample of the wild-collected larvae were identified as belonging to *Cx*. *pipiens* through morphological and PCR analyses, according to the published protocols [31, 33]. Thus, PCR products were run on a 1.5% agarose gel, bands of interest were recovered, and the amplified fragments were purified and sequenced. The obtained sequences were compared with public databases and matched with reference *Cx*. *pipiens* sequences.

### Susceptibility of *Culex pipiens* to *Bti* under laboratory conditions

**Fig 2:**
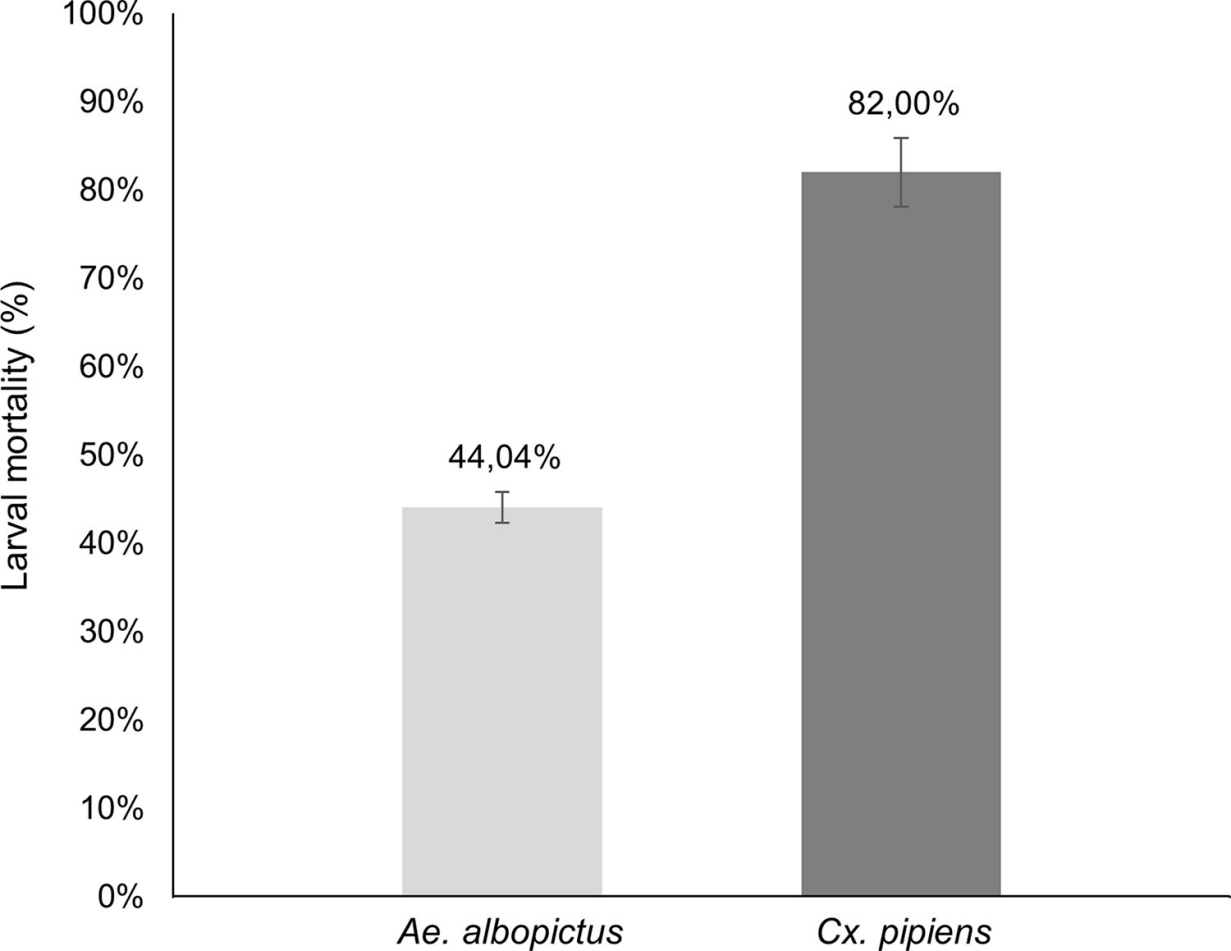
Comparative mortality of *Ae*. *albopictus* (Levate strain) and *Cx pipiens* (Trescore strain) to *Bti*, under laboratory conditions. Fourth instar larvae of both species were exposed for 24 h with the same dose of VectoBac^®^12AS (0.37 mg/L). Data are reported as mean ± standard errors (t(38) = 7.453, *P*<0.0001, Student’s *t* test). Controls mortality was zero.

Bioassays under laboratory conditions were performed to determine whether the commercial insecticide *Bti*-based product VectoBac^®^12AS had similar efficacy on *Ae*. *albopictus* larvae (where it has already been tested, also in rafts) and on *Cx*. *pipiens* larvae, in order to evaluate whether rafts for semi-field experiments could be assembled at the same dosages. Indeed, the LC_50_ dose, effective for *Ae*. *albopictus* larvae, induced a significantly higher mortality in 4^th^ instar *Cx*. *pipiens* larvae after 24 h of exposure (Fig 2) (data in S1 Dataset) and thus MosChito rafts composition that resulted effective on *Ae*. *albopictus* [28] was used in the next experiments.

### Susceptibility of *Culex pipiens* to *Bti* in semi-field bioassays

To evaluate the effectiveness of MosChito rafts on *Cx*. *pipiens*, under conditions that resemble habitats where the tool could potentially be employed for larval control, strains with a different origin were used: the Trescore strain and the wild-collected individuals, from Milan. Following the protocol developed in our previous work [27], MosChito rafts were tested on pools of larvae at different developmental stages.

**Fig 3:**
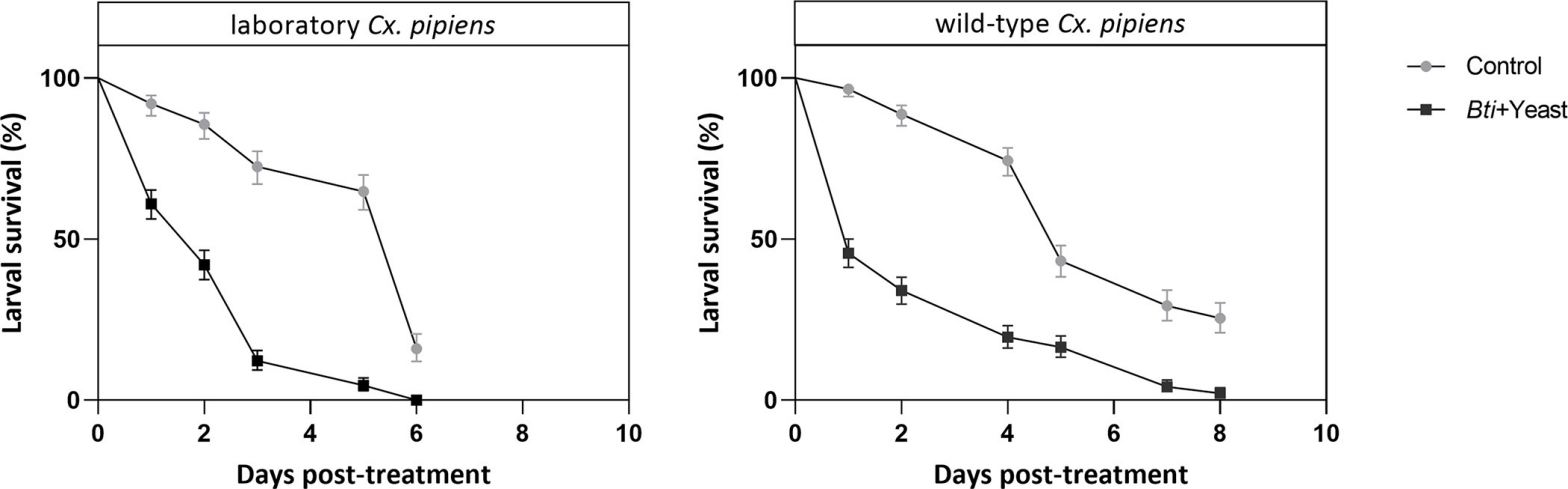
Larval mortality of *Cx*. *pipiens* in bioassays under semi-field conditions. Results obtained with a laboratory strain of *Cx*. *pipiens* (Trescore strain, established less than two years prior to the experiments) (a) or with larvae collected in the field (Milan) and immediately used (b). Larvae of both origins were exposed to control or *Bti*+Y rafts and survival was recorded every 24 h. Results are represented as mean ± standard error (N = 3 independent experiments) (*P* < 0.0001).

For both *Cx*. *pipiens* strains, a mortality rate of more than 50% was observed, as early as the second day of treatment, with a more immediate effect for the wild-collected larvae (over 50% already by the first day) (Fig 3) (data in S1 Dataset). 100% mortality was reached on day six or eight for the laboratory and wild strain, respectively. A concomitant decrease in larval survival was observed on the last days, even for control pools, which probably suffered from the lack of food in the semi-field conditions.

## Discussion

Human coexistence with *Cx*. *pipiens* dates to the end of the Neolithic period, and the historic acquaintance with this mosquito is possibly documented by ancient Egyptian papyri and pharaonic sculptures (as long ago as 2000 B.C.) [1, 34]. In recent decades this mosquito has been recognized as a major threat to human health, due to a series of interrelated factors that have modified its habitat, in parallel with changes in its behavior and in the hosts habits [35, 36]. *Cx*. *pipiens* in origin fed primarily on birds, in warm seasons [37] and in strictly wild and rural areas, but it has now adapted to feed even at lower temperatures and in highly urbanized/anthropized areas on different hosts, including humans [38–40]. Furthermore, urbanization and enhanced commercial trades, combined with gradual climate changes, and rising temperatures, have disrupted the initial balance, reducing distances between the vector, the animal reservoirs of mosquito-borne pathogens and humans, leading to spatial sharing and, consequently, also to pathogen sharing [1, 37]. In particular, in Italy and other European countries, *Cx*. *pipiens* has been found to occupy a wide variety of natural and artificial water containers in wild, rural, and urban areas, often coexisting with the invasive mosquito *Ae*. *albopictus* [29, 30, 41, 42].

The sharing of larval habitats of the major mosquito disease vectors (i.e., *Cx*. *pipiens* and *Ae*. *albopictus*) provides the opportunity to control both species with a single type of intervention. MosChito rafts was developed as an eco-friendly tool for the delivery of bioinsecticides to mosquito larvae [27] and has recently been tested for its efficacy against the Asian tiger mosquito *Ae*. *albopictus* [28]. However, the efficacy of larvicides is variable in different mosquito species [43]. Therefore, with this current study we have determined the efficacy of this tool on *Cx*. *pipiens* larvae. Our results revealed a high efficacy against this species, paving the way towards the use of MosChito rafts in control programs, aimed at the containment of both *Cx*. *pipiens* and other species in the areals where they coexist.

Since laboratory experiments showed an even higher susceptibility to *Bti* of *Cx*. *pipiens* larvae compared to *Ae*. *albopictus*, MosChito rafts used for the present study contained the *Bti* concentration previously applied to *Ae*. *albopictus* in the semi-field tests [28].

Semi-field bioassays showed that *Bti*-containing MosChito rafts cause high mortality rates by the third day of treatment, in both mosquito strains (above 50% by the second day). The slower progression to 100% mortality in *Cx*. *pipiens*, compared to *Ae*. *albopictus*, possibly derives from the different amounts of bioinsecticide ingested, which in turn is due to the different ecology of the two species. Indeed, mosquito species exhibit different behavioral and feeding habits, at both the larval and adult stages, also in relation to the morphology of head and mouthparts [44]. Species from the *Culex* genus have been categorized as “collector-filterers” that feed in the water column and exhibit immobility in presence of food in the water [45, 46], while the *Aedes* species are generally categorized as “collector-gatherers” and “shredders” on detritus. Therefore, we hypothesize that *Aedes* larvae are more likely to directly shred the rafts and thus ingest micro-fragments of MosChito rafts. This above division of mosquitoes into feeding types should be considered with some caution because mosquito larvae have considerable behavioral flexibility in feeding habits, in response to resource availability and sensory stimuli [42, 46, 47]. In particular, the presence of phagostimulant factors, such as nucleic acids or nucleotides from microorganisms or organic surfaces (such as yeast in our case), attracts them to specific feeding areas [48, 49]. *Culex* larvae have previously been observed moving toward the food source, spending time filtering, and beating their mouthparts near/over debris [42, 48]. During the bioassays, we hypothesized that the embedded yeasts were not released from MosChito rafts, as for *Bti*, as previously demonstrated, in [28]. Thus, the strong attractiveness of yeast [50], trapped within the matrix, is not sensed over long distances by the larvae, however, it could be easily enhanced through the addition of other attractive molecules [51]. Moreover, yeasts could be used in the future, in combination with other insecticides (that present a more repellent effect than VectoBac^®^12AS) as bio-factories to produce inhibitory molecules of larval defense systems (i.e., dsRNA, siRNA). The biological control action performed by *Bti* thus would be combined with RNA interference action.

Thus, the use of MosChito rafts is particularly advantageous because the inclusion of *Bti* shields it from sunlight and external agents, that would enhance its degradation. The inclusion of the VectoBac^®^12AS product is permanent and avoids release into the environment, preventing phenomena such as deposition in soil or exposition of mosquito larvae to sublethal doses of bioinsecticide (thus preventing resistance phenomena). Unlike current commercial products that act by dispersion/dissolution, MosChito rafts act in a direct and targeted manner on larvae, in the reproduction site where high doses of *Bti* are ingested, while reducing the concentrations used for the single raft. This translates into a reduction in both cost and environmental impact. Although yeasts do not have an attractive action, they can subsequently be engineered to obtain an additional control method. Furthermore, the dark color, buoyancy, biological and erodible composition make MosChito rafts excellent larval bait.

In conclusion, MosChito rafts represent an environmentally friendly tool for mosquito control, which has been shown to be effective on two larval species that are implicated in the transmission of pathogens affecting humans and animals. A single weapon with a safe approach to control two mosquito species that, despite exhibiting different behavioral characteristics, coexist in the same environment and have a remarkable adaptive capacity, may help to reduce the transmission of different viruses and other pathogens, with a containment of future outbreaks, in Italy and in other countries.

## Supporting information

S1 Dataset Data resulted from multiples bioassay performed in laboratory and semi-field condition and graphical represented in Figs 2 and 3. (XLSX)
